# Data taken from the review article “Radiation and circulatory disease” and used in the associated meta-analysis

**DOI:** 10.1016/j.dib.2016.11.016

**Published:** 2016-11-09

**Authors:** Mark P. Little

**Affiliations:** Radiation Epidemiology Branch, National Cancer Institute, Bethesda, MD 20892-9778, USA

**Keywords:** Circulatory disease, Ionizing radiation, Ischemic heart disease, Stroke, Review

## Abstract

This WinZip archive ‘Combined R, EpiWin & datafiles (rev 2016-9-29).zip’ contains the basic data used in the meta-analysis of doi 10.1016/j.mrrev.2016.07.008 (M.P. Little, 2016) [1], complete with R (R Project version 3.2.2, 2015) [2] code used for meta-regression etc. The basic data is derived from the papers of Mulrooney et al. (D.A. Mulrooney, M.W. Yeazel, T. Kawashima, A.C. Mertens, P. Mitby, M. Stovall, S.S. Donaldson, D.M. Green, C.A. Sklar, L.L. Robison, W.M. Leisenring, 2009) [3], Cutter et al. (D.J. Cutter, M. Schaapveld, S.C. Darby, M. Hauptmann, F.A. van Nimwegen, A.D.G. Krol, C.P.M. Janus, F.E. van Leeuwen, B.M.P. Aleman, 2015) [4] and van Nimwegen et al. (F.A. van Nimwegen, M. Schaapveld, D.J. Cutter, C.P.M. Janus, A.D.G. krol, M. Hauptmann, K. Kooijman, J. Roesink, R. van der Maazen, S.C. Darby, B.M.P. Aleman, F.E. van Leeuwen, 2016) [5], and other data taken from various Tables in the papers summarized in Tables 2–4 of doi 10.1016/j.mrrev.2016.07.008 [1]. The archive also contains R [2] script files that perform the meta-analysis.

**Specifications Table**

**Table:** 

Subject area	Biology
More specific subject area	Ionizing radiation effects on the circulatory system
Type of data	Text data files (also contained in some Excel spreadsheets), additional R [2] and Epicure [6] script and output files(see WinZip archive)
How data was acquired	A systematic review by Little et al. [7] augmented by various auxiliary literature reviews.
Data format	A mixture of text (*.txt), comma separated values (CSV) (*.csv), Excel (*.xlsx), R [2] script files (*.R), Epicure [6] script files (*.gbo) and output files from these (*.txt, *.log).
Experimental factors	The basic data consists of a comma separated values (CSV) file ‘Mulrooney heart data.csv’ and an Excel spreadsheet ‘Mulrooney heart data.xlsx’ containing the same data, comprising summary ERR taken from Table 5 of the paper of Mulrooney et al. [3]. The R script ‘Reanalysis of Mulrooney et al. heart data.R’ fits the necessary weighted ordinary least squares (OLS) models, yielding estimates of ERR/Gy and 95% CI for the four disease endpoints considered by Mulrooney et al. (congestive heart failure, myocardial infarction, pericardial disease, valvular abnormalities); the text output file ‘Reanalysis of Mulrooney et al. heart data.txt’ contains the results of the regressions. The text files ‘Cutter et al. JNCI 2015 valvular heart disease data.txt’ and ‘van Nimwegen et al. JCO 2016 heart disease data.txt’ contain numbers of cases and controls and mean dose by dose group taken from the papers of Cutter et al. [4] and van Nimwegen et al. [5], and the associated Epicure [6] binomial odds ratio scripts ‘Analysis of Cutter et al. valvular data.gbo’ and ‘Analysis of van Nimwegen et al. heart data.gbo’ fit linear binomial odds models. The results of these regressions (in the text files ‘Analysis of Cutter et al. valvular data.log’ and ‘Analysis of van Nimwegen et al. heart data.log’) along with much other data taken from various Tables in the papers summarized in Tables 2-4 of Little [1] is then incorporated in the Excel spreadsheet ‘Cardiovascular disease risks (various cohorts).xlsx’. Summary data from this spreadsheet is given in the CSV file ‘Mut Res Reviews data.csv’. This file is the basis of all the meta analysis performed in the main paper by Little [1]. The R [2] script files that do this are ׳Meta analysis of circulatory disease data max likelihood Mayak mortality.R׳, ׳Meta analysis of ischemic heart disease data Mayak mortality.R׳, ׳Meta analysis of cerebrovascular disease data Mayak mortality.R׳, ׳Meta analysis of ischemic heart disease data.R׳, ׳Meta analysis of circulatory disease data max likelihood.R׳, ׳Meta analysis of cerebrovascular disease data.R׳, ׳Meta analysis of circulatory disease data.R׳, and the associated text output files, as used in the main paper, are ׳Meta analysis of circulatory disease data max likelihood Mayak mortality.txt׳, ׳Meta analysis of ischemic heart disease data Mayak mortality.txt׳, ׳Meta analysis of cerebrovascular disease data Mayak mortality.txt׳, ׳Meta analysis of ischemic heart disease data.txt׳, ׳Meta analysis of circulatory disease data max likelihood.txt׳, ׳Meta analysis of cerebrovascular disease data.txt׳, ׳Meta analysis of circulatory disease data.txt׳, respectively.
Experimental features	The data given in the CSV file ‘Mut Res Reviews data.csv’ that is used for all the meta-analyses in the paper is all comma separated text, and is derived in the manner explained above.
Data source location	NA
Data accessibility	Data is with this article

**Value of the data**

Data can be used to duplicate and extend meta-analysis of radiation and circulatory disease of Little [1].

## 1 Data

The basic data consists of a comma separated values (CSV) file ‘Mulrooney heart data.csv’ and an Excel spreadsheet ‘Mulrooney heart data.xlsx’ containing the same data, comprising summary ERR taken from Table 5 of the paper of Mulrooney et al. [3]. The R [2] script ‘Reanalysis of Mulrooney et al. heart data.R’ fits the necessary weighted ordinary least squares (OLS) models, yielding estimates of ERR/Gy and 95% CI for the four disease endpoints considered by Mulrooney et al. (congestive heart failure, myocardial infarction, pericardial disease, valvular abnormalities); the text output file ‘Reanalysis of Mulrooney et al. heart data.txt’ contains the results of the regressions. The text files ‘Cutter et al. JNCI 2015 valvular heart disease data.txt’ and ‘van Nimwegen et al. JCO 2016 heart disease data.txt’ contain numbers of cases and controls and mean dose by dose group taken from the papers of Cutter et al. [4] and van Nimwegen et al. [5], and the associated Epicure [6] binomial odds ratio scripts ‘Analysis of Cutter et al. valvular data.gbo’ and ‘Analysis of van Nimwegen et al. heart data.gbo’ fit linear binomial odds models. The results of these regressions (in the text files ‘Analysis of Cutter et al. valvular data.log’ and ‘Analysis of van Nimwegen et al. heart data.log’) along with other data taken from various Tables in the papers summarized in Tables 2–4 of Little [1] is then incorporated in the Excel spreadsheet ‘Cardiovascular disease risks (various cohorts).xlsx’. Summary data from this spreadsheet is given in the CSV file ‘Mut Res Reviews data.csv’. This file is the basis of all the meta analysis performed in the main paper (doi 10.1016/j.mrrev.2016.07.008 [1]). The R [2] script files that do this are ׳Meta analysis of circulatory disease data max likelihood Mayak mortality.R׳, ׳Meta analysis of ischemic heart disease data Mayak mortality.R׳, ׳Meta analysis of cerebrovascular disease data Mayak mortality.R׳, ׳Meta analysis of ischemic heart disease data.R׳, ׳Meta analysis of circulatory disease data max likelihood.R׳, ׳Meta analysis of cerebrovascular disease data.R׳, ׳Meta analysis of circulatory disease data.R׳, and the associated text output files, as used in the main paper, are ׳Meta analysis of circulatory disease data max likelihood Mayak mortality.txt׳, ׳Meta analysis of ischemic heart disease data Mayak mortality.txt׳, ׳Meta analysis of cerebrovascular disease data Mayak mortality.txt׳, ׳Meta analysis of ischemic heart disease data.txt׳, ׳Meta analysis of circulatory disease data max likelihood.txt׳, ׳Meta analysis of cerebrovascular disease data.txt׳, ׳Meta analysis of circulatory disease data.txt׳, respectively.

## 2 Experimental design, materials and methods

As outlined above.
