# Supplementary material for: Data taken from the review article “Radiation and circulatory disease” and used in the associated meta-analysis
Source: Data Brief. 2016 Nov 9;9:1024–7. doi: 10.1016/j.dib.2016.11.016 (PMC5123126; doi:10.1016/j.dib.2016.11.016)
Supplement: Supplementary file 1 — Supplementary material [file mmc1.doc]

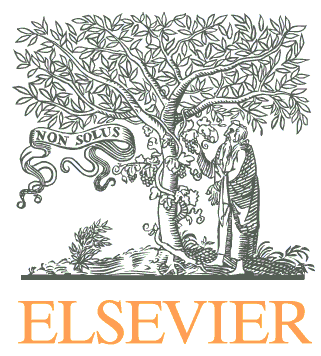


***Mutation Research-Reviews in Mutation Research***

**Conflict of Interest Policy**

Manuscript number (if applicable): **DIB-D-16-00660**

Article Title: **Data taken from the review article “Radiation and circulatory disease” and used in the associated meta-analysis**

Author name: **Mark P. Little**

**Declarations**

***Mutation Research-Reviews in Mutation Research***  requires that all authors sign a declaration of conflicting interests. If you have nothing to declare in any of these categories then this should be stated.

**Conflict of Interest**

A conflicting interest exists when professional judgement concerning a primary interest (such as patient’s welfare or the validity of research) may be influenced by a secondary interest (such as financial gain or personal rivalry). It may arise for the authors when they have financial interest that may influence their interpretation of their results or those of others. Examples of potential conflicts of interest include employment, consultancies, stock ownership, honoraria, paid expert testimony, patent applications/registrations, and grants or other funding.

**Please state any competing interests**

| I have no competing interests. |
| --- |

**Funding Source**
All sources of funding should also be acknowledged and you should declare any involvement of study sponsors in the study design; collection, analysis and interpretation of data; the writing of the manuscript; the decision to submit the manuscript for publication. If the study sponsors had no such involvement, this should be stated.

**Please state any sources of funding for your research**

| The research was supported by the Intramural Research Program of the Division of Cancer Epidemiology and Genetics, National Institutes of Health. |
| --- |

**Signature** (a scanned signature is acceptable, **Print name**

but each author must sign)

_______________ Mark Little_____________________
